# An automated approach for predicting glioma grade and survival of LGG patients using CNN and radiomics

**DOI:** 10.3389/fonc.2022.969907

**Published:** 2022-08-12

**Authors:** Chenan Xu, Yuanyuan Peng, Weifang Zhu, Zhongyue Chen, Jianrui Li, Wenhao Tan, Zhiqiang Zhang, Xinjian Chen

**Affiliations:** ^1^ State Key Laboratory of Radiation Medicine and Protection, Collaborative Innovation Center of Radiological Medicine of Jiangsu Higher Education Institutions, and School for Radiological and Interdisciplinary Sciences (RAD-X), Soochow University, Suzhou, China; ^2^ School of Electronics and Information Engineering and Medical Image Processing, Analysis and Visualization Lab, Soochow University, Suzhou, China; ^3^ Department of Diagnostic Radiology, Affiliated Jinling Hospital, Medical School of Nanjing University, Nanjing, China; ^4^ State Key Laboratory of Analytical Chemistry for Life Science, Nanjing University, Nanjing, China

**Keywords:** automatic diagnosis, glioma grade, survival of lower-grade glioma, convolutional neural network, radiomics

## Abstract

**Objectives:**

To develop and validate an efficient and automatically computational approach for stratifying glioma grades and predicting survival of lower-grade glioma (LGG) patients using an integration of state-of-the-art convolutional neural network (CNN) and radiomics.

**Method:**

This retrospective study reviewed 470 preoperative MR images of glioma from BraTs public dataset (n=269) and Jinling hospital (n=201). A fully automated pipeline incorporating tumor segmentation and grading was developed, which can avoid variability and subjectivity of manual segmentations. First, an integrated approach by fusing CNN features and radiomics features was employed to stratify glioma grades. Then, a deep-radiomics signature based on the integrated approach for predicting survival of LGG patients was developed and subsequently validated in an independent cohort.

**Results:**

The performance of tumor segmentation achieved a Dice coefficient of 0.81. The intraclass correlation coefficients (ICCs) of the radiomics features between the segmentation network and physicians were all over 0.75. The performance of glioma grading based on integrated approach achieved the area under the curve (AUC) of 0.958, showing the effectiveness of the integrated approach. The multivariable Cox regression results demonstrated that the deep-radiomics signature remained an independent prognostic factor and the integrated nomogram showed significantly better performance than the clinical nomogram in predicting overall survival of LGG patients (C-index: 0.865 vs. 0.796, *P*=0.005).

**Conclusion:**

The proposed integrated approach can be noninvasively and efficiently applied in prediction of gliomas grade and survival. Moreover, our fully automated pipeline successfully achieved computerized segmentation instead of manual segmentation, which shows the potential to be a reproducible approach in clinical practice.

## Introduction

Glioma is one of the most common malignant tumors of the central nervous system, which is characterized by invasive growth, contributing to high mortality and recurrence rates (1–4). According to the standard classification of the World Health Organization (WHO), glioma can be subdivided by their malignancy into lower-grade glioma (grade II/III, LGG) and higher-grade glioma (grade IV, HGG) (5, 6). Gliomas have a wide range of prognoses depending on the WHO grade, with a median survival of 14 months for HGG patients and is longer in LGG patients as survival times of up to 10 years and longer can be observed in Refs (7–9). LGG patients may have a longer lifespan than HGG patients, and similarly, LGG patients may develop into HGG patients over time (10). Although the recent WHO recommendations mainly rely on tumor genomic profile (2), histologic grading is a vital phenotypic measure. Nowadays, pathological biopsy has been used for histological grading, pathologists usually select the highest component from the histopathology samples to predict the overall tumor grade, which inevitably brings risks (11). Therefore, a non-invasive and reproducible technique for glioma grading and prognosis evaluation is of scientific and clinical value, which is helpful to make treatment plans and preoperative prognostic analysis.

Among the numerous imaging techniques, MRI is the most widely used imaging modality for the diagnosis and prognosis of gliomas. It does not use ionizing radiation and provides versatile contrast for various brain tissues (12), which can represent the signal difference of many different gray levels while the naked eyes can only distinguish 16 gray levels. Therefore, comprehensive information has not been fully utilized to analysis tumor heterogeneity in previous clinical practice and is necessary to analysis by emerging artificial intelligence technology. Radiomics is an emerging methodology that can extract comprehensive and quantitative features from medical images to predict tumor type, grade, genomic and transcriptomic subtypes (13–16). However, most studies were based on manually delineating tumor regions and then using data mining algorithms such as machine learning to analysis (13, 15), which may inevitably lead to time-consuming and errors from different raters. It may be a leap forward in the field of computer-aided diagnosis if this hurdle can be resolved. Therefore, it is important to develop an automated approach to objectively and accurately predict the glioma grade and survival of LGG patients.

Convolutional neural network (CNN), a type of deep learning architecture, can exploit high-dimensional numeric information from images by learning relevant features directly from image signal intensities, which have been proven to be particularly useful in medical image analysis (17). Although several studies have employed MRI-based CNN models to stratify glioma grades (18–26), few studies have applied CNN to survival analysis, which may be due to the great differences in individual survival time and the insufficiency of patients for CNN training. In addition, among the studies of stratifying tumor grades using CNN, it is mostly based on 2D MRI images (20–22, 24–26), which inevitably loses the 3D spatial contextual information. In particular, several studies have applied radiomics features to predict prognosis of glioma (10, 27–29). For example, Bae S et al. demonstrated that the radiomics features extracted from multiparametric MRI have prognostic value (30). In addition, among existing studies used CNN for prognostic analysis (31–33), and most of these studies predict overall survival (OS) of glioblastoma patients. As for LGG, although most of the patients survive a long time, there is still a large subset of patients who have a very short lifespan (34, 35). To mitigate this limitation, we analyzed the prognostic factors of LGG patients and developed a reproductive model to identify LGG patients with poor prognosis in this study.

The purpose of this study was to propose an automated approach for predicting glioma grade and overall survival for LGG patients. First, we developed a CNN for the automated tumor segmentation. Then, we developed a classifier for grading that can integrate features extracted from 3D CNN network and radiomics features. In addition, we constructed a deep-radiomics signature using CNN and radiomics features, and validated the hypothesis that the deep-radiomics signature was an independent prognostic factor of LGG patients. Finally, a prognostic nomogram was developed to predict the OS of LGG patients and validated in an independently cohort.

## Materials and methods

This retrospective study was approved by the Institutional Review Board of Jinling Hospital, Medical School of Nanjing University. Informed consent was waived because of the use of retrospective image data.

### Data collection

This retrospective multicenter study enrolled patients who were diagnosed as glioma. All patients were included in this study by the following criteria: (1) both MRI sequences were available before any treatment, (2) pathological results based on WHO classification were available, (3) MRI images were provided with T1, T1-Gd, T2 weighted sequences, and (4) All MRI images have diagnostic image quality.

The first dataset is BraTS 2020 data (36–38) (denoted as center A), which includes 293 HGG patients and 76 LGG patients. All BraTS multimodal images were pre-operative scans and have been segmented manually, by one to four raters according to the same annotation protocol, with annotations approved by experienced neuro-radiologists. All the images have been skull-stripped, co-registered to the same anatomical template and interpolated to the same resolution (1×1×1 mm³).

The second dataset (denoted as center B) is from Jinling Hospital including 89 HGG patients and 114 LGG patients. MRI acquisition parameters are listed in **Supplementary Material S1**. In correspondence with the first dataset, a preprocessing pipeline including bias correction using N4ITK method (39), skull stripe, registration, and intensity normalization was applied to all MRI images. Each modality images were aligned into the same geometric space using the General Registrations (BRAINS) Toolbox in 3D Slicer (version 4.11, www.slicer.org), and normalized independently by subtracting the mean and dividing by the standard deviation of the brain region. After registration, tumor regions of interest (ROI) were segmented slice by slice using ITK-SNAP software (version 3.6.0-RC1; http://www.itk-snap.org) on T1-Gd sequence. The ROI segmentation was performed by two radiologists with more than 10 years of experience in neuroimaging. All of them were blinded to the clinical information of the patients. The ICCs were used to evaluate the consistency of tumor extraction by different physicians.

For tumor segmentation and grading, a total of 470 patients were enrolled and they were randomly divided into the training cohort (n = 284), validation cohort (n = 93) and test cohort (n = 93), with a ratio of 6:2:2.

For LGG survival analysis, the survival information of 61 LGG patients from April 2012 to June 2015 was published in first dataset. The second dataset is an independent validation dataset composed of 112 LGG patients in Jinling Hospital of Nanjing University from January 2018 to October 2021. OS of all patients in two institution was calculated from the data of diagnosis until death or last follow-up visit. The patient enrollment pathway was presented in **Figure S1**.

### Tumor segmentation and grading

Tumor segmentation was used as a preparation for automated tumor classification, which was implemented by CNN. For the network architecture, we have made a couple of changes to the original 3D U-net proposed by Isensee et al. (2019) (40), which mainly consists of encoder and decoder and is shown in **Figure S2**. The top encoder is called context path, which can generate more abstract image features for feature extraction with the deepening of layers. At the bottom, the decoder is called location path, which can gradually increase the resolution by upsampling high-semantic features. In addition, skip connections are used to combine the high spatial resolution features in the context path with the low spatial resolution features in the localization path to improve the segmentation performance. Since the direct use of convolution cannot achieve good performance in feature extraction, recombination blocks are used to enhance the ability of feature extraction. As for recombination blocks, the number of channels is increased by convolution (kernel size:1×1×1, number of convolution kernels: 64). Then, the convolution is followed by a batch normalization and a ReLu activation function as well as a convolution (kernel size:3×3×3). Next, a squeeze-and-excitation block (SE Block) (41) is adopted to improve the refinement ability of feature information. Specifically, the encoder consists of a series of modules, including four layers of recombination blocks, each of which is followed by a max-pooling layer. The decoder consists of four upsampling blocks corresponding to the encoder, and each block contains a transposed convolution (kernel size:2×2×2, stride=2) and a recombination block. The last layer of the network is a softmax activation function that produces tumor segmentation results. Due to the limitation of computational resources, the batch size was set as 1 and total training epochs were set as 200. Training was started with an initial learning rate of 5 ×10^-4^. Learning rate was reduced by half after 10 epochs if the validation loss did not improve. The loss function in this study was a joint loss, which consisted of dice loss and binary cross-entropy (BCE) loss. The total loss function combined was defined as follow:


(1) $$L_{total}=L_{Dice}+L_{BCE}$$

where,


(2) $$L_{Dice}=1-\frac{2\left|X*Y\right|}{\left|X\right|+\left|Y\right|}$$

(3) $$L_{BCE}=\;-\sum_{h,w}\left(1-Y\right)\log\left(1-X\right)+Ylog\left(X\right)$$

where X and Y were the segmentation results and the corresponding ground truth, h and w were the coordinates of the pixel in X and Y.

**Figure 1:**
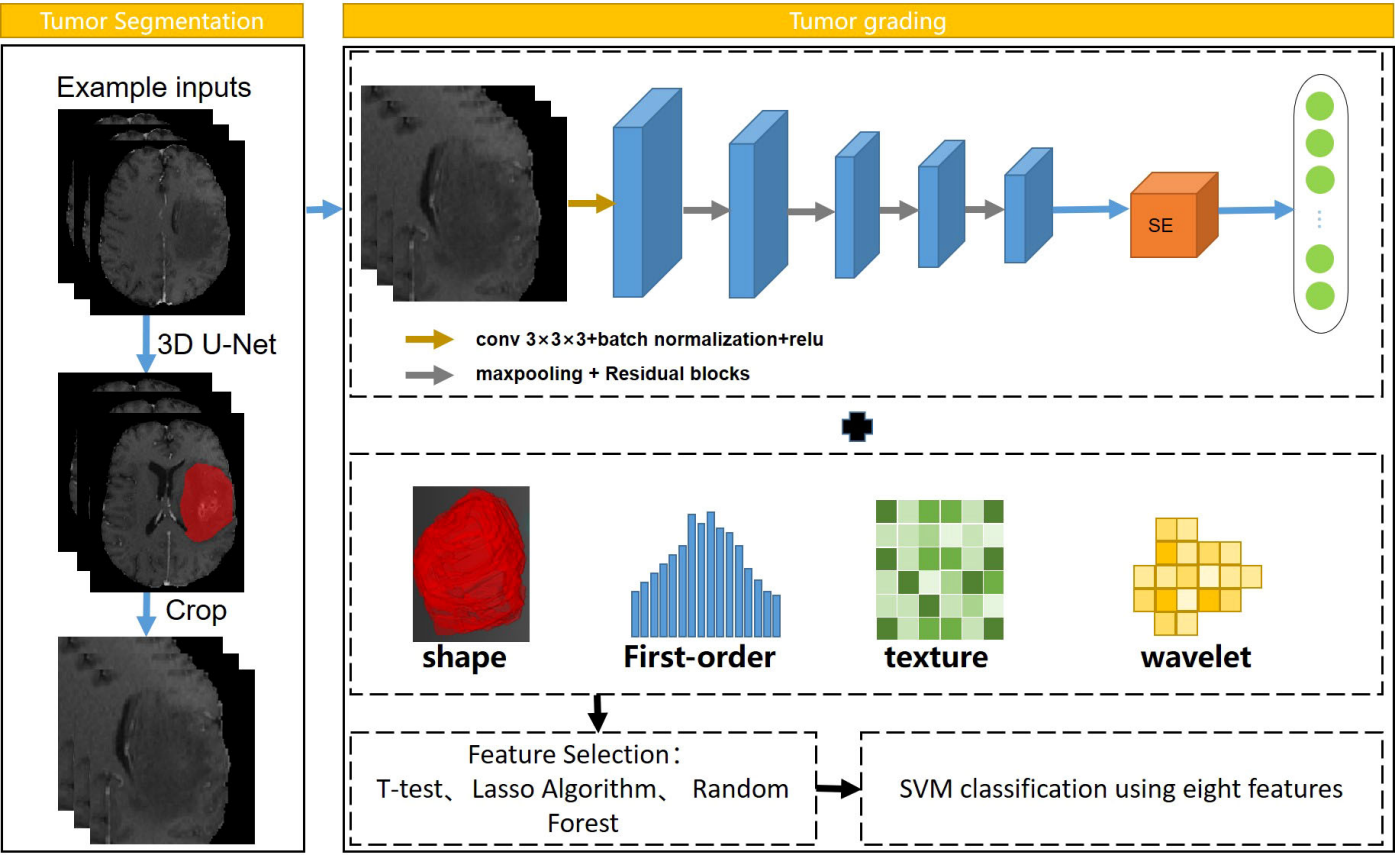
Workflow of automatic tumor grading. Resnet18 was used to extract CNN features, and SVM was selected as the classifier.

In addition, the coordinate cropping method is used to traverse all the segmentation results and obtain the coordinate information of the tumor on the MRI image and select the slice with the largest area as the size of the patches. Then, the corresponding patches were cropped at the center of the tumor on the MRI image to ensure that the tumor area is all in the patches. Both radiomics and deep learning belong to the field of machine learning. Radiomics extracts high-throughput features using traditional algorithms, while deep learning directly uses CNN to extract highly generalized features. The workflow of tumor grading is provided in **Figure 1**.

All radiomic features were extracted by Pyradiomics (https://pyradiomics.readthedocs.io). We used the open-source “pyradiomics” package to extract the radiomics features of MRI images (42). More detailed information about the radiomics features can be found in **Supplementary Material S2** (**Table S1**). It is noteworthy that we attempted to use the result of segmentation network as the mask in radiomics feature extraction. ICCs (43) were used to evaluate the consistency of tumor extraction between the segmentation network and the physicians. To avoid feature redundancy caused by the simultaneous use of radiomics features from three sequences, the grading experiments conducted using radiomics method in three sequences of MRI images respectively.

For deep CNN features, we used ResNet18 (44) as the backbone of 3D CNN model. The four residual blocks in the ResNet18 backbone, including convolutional and pooling layers, were implemented in 3D. In addition, a SE block which adaptively recalibrated channel-wise feature responses by explicitly modelling interdependencies between channels, was added after the fourth residual block. This block was designed to improve the performance of the model by exploring the relationship of feature maps between different channels. The tumor patches obtained by the coordinate cropping method in the tumor segmentation experiment were firstly fed into the 3D CNN network. Then, a total of 512 deep features obtained by the average pooling layer after the fourth residual block from each patient. The patch size of the input was set to 96×128×96. The batch size and epochs of the CNN model training process were set to 8 and 200, respectively. Finally, we combined radiomics features with CNN features through a concatenation operation to obtain a total of 1300 features for each patient.

For machine learning models, in the process of dimensionality reduction for a large number of features, the features related to the results should be retained as much as possible, and the information carried by irrelevant features and redundant features should be removed to prevent overfitting of the classification model. The Z-Score normalization was used on the features of the training cohort, which was aimed to uniformly convert the magnitudes of different features into the same magnitude to ensure that comparability between data. The Levene’s test (45) and the least absolute shrinkage and selection operator (LASSO) were used to select subsets of features. Next, the random forest (46) was utilized to compare the contribution of different features according to the importance of the features. The number of trees was set to 100, and finally, eight features were acquired. The support vector machine (SVM) was used as classifier due to its good performance (47). In this process, the GridSearchCV algorithm (48) was used to search the optimal penalty factor C and the gamma parameters for the SVM model (49), where the former determined the tolerance of the samples, and the later determined the number of support vectors.

### Building of deep-radiomics signature

**Figure 2:**
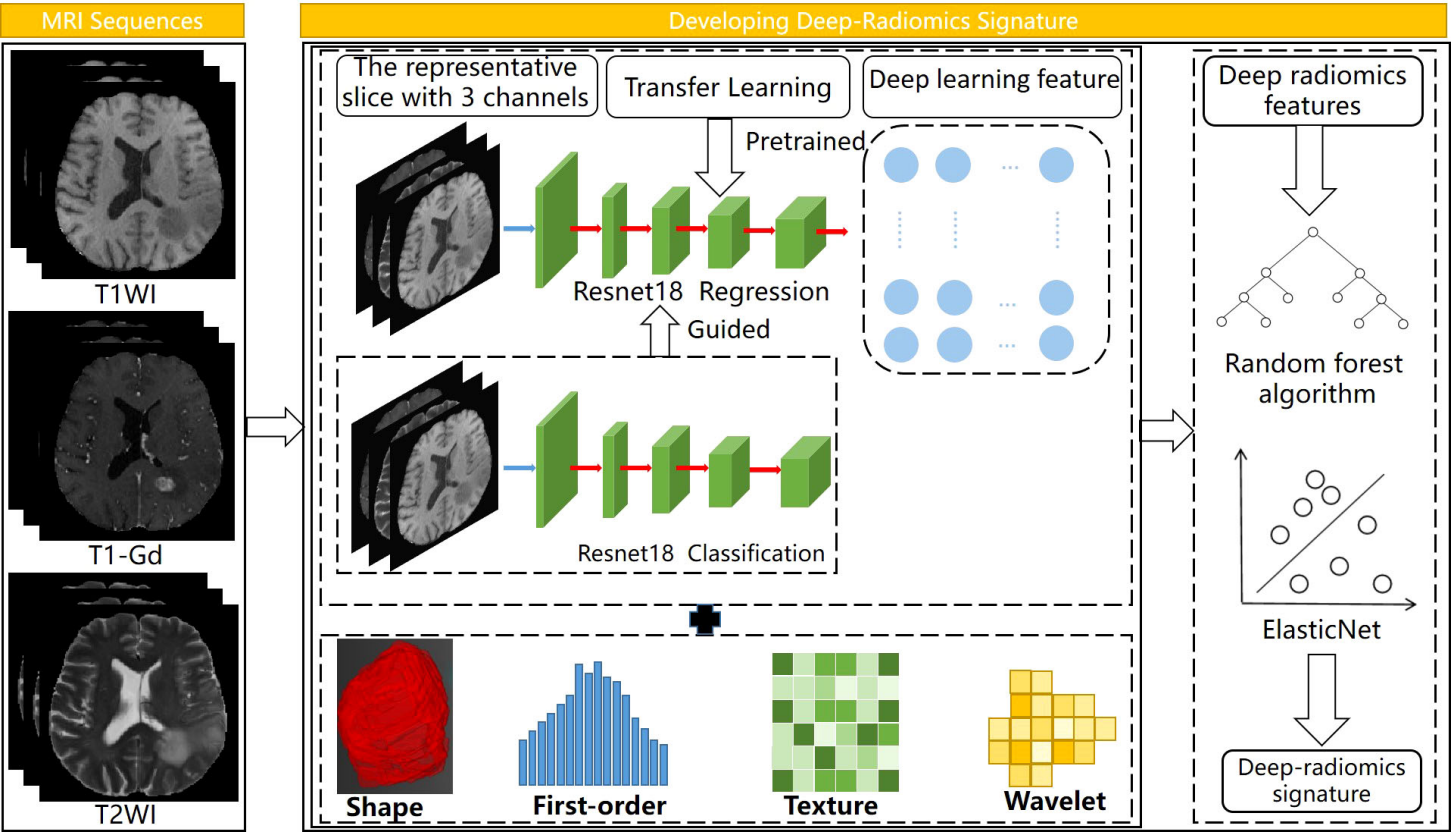
Workflow of developing deep-radiomics signature, the regression network was guided by the classification network. Elastic Net was used to develop the deep radiomics signature.

For the survival analysis of LGG patients, we developed a deep-radiomics signature and conducted a multicenter analysis. Cox regression models were established for the survival factors of patients in center B, and independently validated in center A. The workflow of this part is shown in **Figure 2**. Firstly, we developed three radiomics models for predicting tumor prognosis based on three modalities sequences to select the best sequence to reflect potential tumor prognosis information. For the convenience of modeling, the patients that lower than the median OS were assigned as unfavorable prognosis, and the remaining patients were assigned as favorable prognosis (50). The Levene’s test and random forest algorithm were used in feature selection, and SVM was selected as the classifier. Then we selected the sequence with the best performance to extract radiomics features for building the deep-radiomics signature. A total of 788 radiomics features were extracted as radiomics features for each patient.

In CNN, ResNet18 was used as the backbone network. We designed a classification-guided prediction strategy to simultaneously perform the dual tasks of grading LGG patients and regressing survival time on the network. The weights of the two tasks were shared during the training process. Through back propagation, dual-task learning could complement each other by sharing information and improved each other’s performance. However, deep learning models required a lot of data for training. To deal with the limitation of the small amount of data in survival analysis, the transfer learning method was introduced to improve the robustness and generalization ability of the model. We selected the slice with the largest tumor area and used the weights pre-trained by ImageNet (17) to assist our model to reduce a lot of computational cost and make the model converge as soon as possible. Considering the MRI images were single-channel images and the images in ImageNet are three-channel, we fused T1, T2, and T1-Gd weighted sequences on the channel so that the network could obtained more information. The image size of the input was 3×240×240 and the batch size and epochs during training process were set to 8 and 200, respectively. In total, 512 CNN features were extracted as deep learning features for each patient.

Finally, multidimensional features obtained by fusing CNN features and radiomics features were used for further analysis. All features were normalized by Z-Score normalization. In feature selection, the random forest algorithm was used to calculate the correlation between features and survival time, and ten candidate features were selected. Next, Elastic Net (51) was used to determine feature weights and develop the deep-radiomics learning signature.

### Building of prognostic nomogram

The candidate prognostic indicators included age, sex, tumor location, WHO grade, laterality, histologic type and contrast enhancement. To evaluate the potential association between the deep-radiomics signature and OS, the median value of signatures was determined as a threshold to divide patients into high-risk group and low-risk group by using the X-tile software (50). The Kaplan-Meier (KM) method was used to describe the survival rates of the two groups, and the log-rank test was utilized to compare the differences in the KM curves.

First, an integrated nomogram was developed in the training cohort. Clinical factors and deep-radiomics signature were used as candidate prognostic factors, and Cox regression models were used for univariate and multivariate analyses of these factors. In univariate and multivariate analyses, factors were dropped if *P*-values of them were more than 0.05. Those independent factors after the multivariate analysis were used to build integrated nomogram. For fair comparison, a clinical nomogram was developed using clinical factors. Then, both the integrated nomogram and clinical nomogram were validated on the internal and external validation cohorts. The discrimination performance was measured using C-index. To compare the difference between the predicted survival of the nomogram and the observed survival, the calibration curve plots were used to evaluate the calibration results.

### Statistical analysis

In statistical analysis, continuous variables and categorical variables were compared by Mann-Whitney U test and Chi-square. The automated grading pipeline was conducted by using python 3.7.3. As for prognostic analysis, the survival model building was implemented by using R software, version 4.2.0 (http://www.R-project.org). The comparison of the AUC and the C-index were implemented by using the Delong test. All statistical tests were two-sided and variables with *p*<0.05 were regarded significant.

## Results

### Clinical characteristics of the patients

For tumor grading, 470 patients were randomly divided into the training cohort (177HGGs, 107LGGs, median age 58 years), the validation cohort (58HGGs, 35LGGs, median age 55 years), and the test cohort (58HGGs, 35LGGs, median age 56 years). Since the validation cohort was used to supervise training and represent the performance of the model during training, statistical analysis was performed between the combination of training cohort with validation cohort and the test cohort and the results showed that there were no significant differences in age or tumor grade between the two cohorts (age: *P*=0.539, grade: *P*=0.996).

In the survival analysis of LGG patients, 112 LGG patients from Jinling Hospital were assigned to the training cohort, and an independent validation was performed on 61patients from the BraTs dataset. No significant differences were found between the training cohort and the validation cohort in terms of gender, age, grade, laterality, tumor location, pathological type, OS, and contrast enhancement in MRI (*P*>0.05). **Table S2** summarized the clinical characteristics of the training and validation cohorts.

### Performance of tumor segmentation and grading

The segmentation network was trained from scratch in an image-to-image fashion. The segmentation performance of the segmentation network was based on the Dice coefficient which was 0.81. The interobserver ICCs ranged from 0.803 to 0.871, indicating favorable consistency of tumor ROI extracted by different physicians. The ICCs between segmentation CNN and physicians ranged from 0.793 to 0.901. In radiomics grading models, the radiomics model developed based on T1-Gd sequence yielded the highest AUC, which was 0.894 (95% CI: 0.892-0.896). The detailed quantitative results including the AUC, accuracy, sensitivity, and precision of the models were shown in **Table S3**. Therefore, the radiomics features of T1-Gd sequence was adopted in the next analysis. Ablation experiments were conducted to demonstrate the effectiveness of the proposed method. In the models based on integrated approach, the model with SE attention block based on patches cropped by segmentation results achieved the best performance with an AUC of 0.958 (95% CI: 0.955–0.961), accuracy of 0.941, sensitivity of 0.971, precision of 0.931. The experimental results were shown in **Table 1** (**Figure S3**).

**Table 1:** The experimental results of tumor grading using combination of CNN and radiomics methods.

Methods	AUC (95% CI)	Accuracy	Sensitivity	Precision
Baseline (MRI patches)	0.945 (0.938, 0.952)	0.922	0.923	0.962
Baseline + SE module (MRI patches)	0.958 (0.955,0.961)	0.941	0.971	0.931
Baseline (complete MRI)	0.904 (0.898, 0.910)	0.871	0.912	0.891
Baseline + SE module (complete MRI)	0.932 (0.928, 0.936)	0.881	0.901	0.872

To prove the benefits of using this integrated approach, comparison experiments were conducted using the CNN method. MRI images were fed as the input of the ResNet18 model, which was consistent with the CNN features extraction part of the integrated approach. A total of 512 CNN features were obtained after the fourth residual block, and then through two fully connected layers, the final classification result of CNN model can be obtained. The parameters of the CNN model in the comparison experiment, such as batch size and epoch, were consistent with those in the integrated approach. As can be seen from **Table 2**, the model trained on a CNN with SE block based on the patches achieved the best performance, with an AUC of 0.852 (95% CI: 0.846–0.858), accuracy of 0.874, sensitivity of 0.931, precision of 0.841, which suggested the integrated approach outperformed the CNN method (AUC: 0.958 vs. 0.852, *P*=0.013).

**Table 2:** The experimental results of grading using CNN methods.

CNN methods	AUC (95% CI)	Accuracy	Sensitivity	Precision
Baseline (MRI patches)	0.846 (0.839, 0.855)	0.868	0.912	0.860
Baseline + SE module (MRI patches)	0.852 (0.846,0.858)	0.874	0.931	0.841
Baseline (complete MRI)	0.801 (0.794, 0.811)	0.822	0.873	0.797
Baseline + SE module (complete MRI)	0.794 (0.786, 0.802)	0.833	0.908	0.804

### Building and validation of deep-radiomics signature

In the experiments of predicting tumor prognosis using radiomics method based on three modalities sequences, the radiomics model based on T1-Gd sequence yielded the highest AUC, which was 0.783 (95% CI: 0.774-0.792). The radiomics model based on T1WI and T2WI sequences yielded AUC of 0.754 (95% CI: 0.726–0.781) and 0.638 (95% CI: 0.606–0.670), respectively. Therefore, the radiomics features extracted based on T1-Gd sequence were adopted in the next analysis.

**Figure 3:**
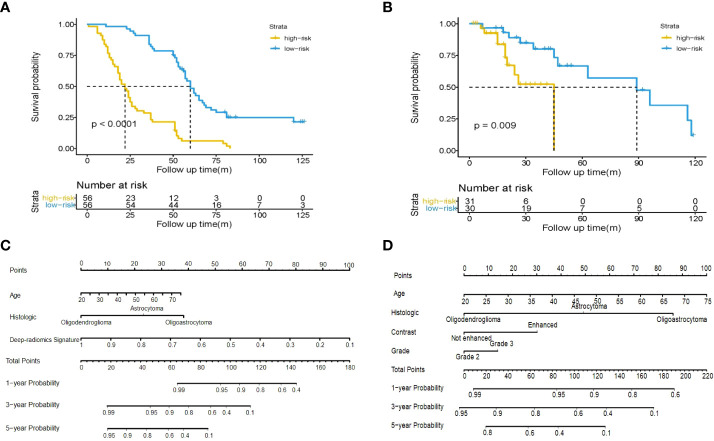
Kaplan-Meier plot for OS of patients stratified by the median value of deep-radiomics signature. Significantly favorable survival in low-risk patients compared to high-risk patients was shown in the training cohort **(A)** and the validation cohort **(B)**. Use of the developed integrated nomogram **(C)** and clinical nomogram **(D)** estimated the OS for LGG.

After feature fusion and feature selection, there were 10 image features including 6 features extracted from CNN and 4 features extracted from radiomics, the names of the features and corresponding weights were detailed in **Table S4**. The median value of the deep-radiomics signature in the training cohort was used as the cutoff for stratifying patients into high-risk group (deep-radiomics signature< 0.475) and low-risk group (deep-radiomics signature ≥ 0.475). The patients in the low-risk group achieved better OS than the high-risk group (*P*< 0.001). Then, the same cutoff value was applied to the validation cohort and yielded similar result. The Kaplan–Meier curves of high-risk and low-risk groups in the training and validation cohorts were illustrated in **Figures 3A, B**, which demonstrated deep-radiomics signature was an independent prognostic factor.

### Construction of clinical nomogram and integrated nomogram

The age, grade, histologic type, contrast enhancement and deep-radiomics signature were identified as prognostic factors correlated to OS in the univariate analysis. Then, a multivariable Cox regression model was developed using statistically significant factors after the univariate Cox regression model. The grade and contrast enhancement were dropped because they failed to remain as independent prognostic factors (**Table 3**). An integrated nomogram was built using prognostic factors (**Figure 3C**). When establishing the model based on clinical factors, age, grade, histologic type and contrast enhancement were identified as independent prognostic factors and a clinical nomogram was developed (**Figure 3D**; **Table 4**).

**Table 3:** Multivariate Cox regression analyses for overall survival in the training and validation cohorts of patients with LGG.

Characteristics	Training cohort		Validation cohort	
	HR (95% CI)	*P*	HR (95% CI)	*P*
Age	1.03 (1.01-1.06)	0.003	1.08 (1.03-1.14)	0.003
Grade III vs. II;	1.96 (1.49-2.43)	0.188	3.77 (0.88 -6.66)	0.073
Histologic type, O vs. A	0.31 (0.06-0.56)	<0.001	0.17 (0.04-0.30)	0.020
Histologic type, OA vs. A	2.27 (0.97-3.57)	0.060	1.44 (0.23-2.65)	0.699
Contrast enhancement vs. not enhanced	1.55 (0.86-2.24)	0.140	3.65 (1.85-5.45)	0.059
Signature above the median vs. below the median	5.69 (4.39-6.99)	<0.001	6.28 (4.32-8.23)	<0.001

HR, Hazard Ratio; 95% CI, 95% Confidence Interval; O, Oligodendroglioma; A, Astrocytoma; OA, Oligoastrocytoma; Signature, Deep- radiomics signature.

**Table 4:** Multivariate Cox regression analyses of clinical data for overall survival in the training and validation cohorts of patients with LGG.

Characteristic	Training cohort		Validation cohort	
	HR (95% CI)	*P*	HR (95% CI)	*P*
Age	1.04 (1.02-1.06)	<0.001	1.08 (1.03-1.14)	0.003
Grade III vs. II;	1.85 (1.49-2.21)	0.026	5.26 (1.95-8.57)	0.009
Histologic type, O vs. A	0.35 (0.19-0.51)	<0.001	0.17 (0.04-0.30)	0.011
Histologic type, OA vs. A	2.22 (0.97-3.47)	0.059	2.38 (1.40-3.36)	0.017
Contrast enhancement vs. Not enhanced	3.26 (2.59-3.93)	0.032	5.57 (3.21-7.93)	0.002

HR, Hazard Ratio; 95% CI, 95% Confidence Interval; O, Oligodendroglioma; A, Astrocytoma; OA, Oligoastrocytoma.

In the internal validation, the integrated nomogram for predicting survival at 1, 3, or 5 years achieved a C-index (0.873, 95% CI: 0.840-0.906) and was outperformed than that of the clinical nomogram (0.770, 95% CI: 0.720-0.820). Consistent results can be found in the external validation, where the C-index of the integrated nomogram (0.865, 95% CI: 0.851-0.879) remained significantly higher than the clinical nomogram (0.796, 95% CI: 0.787-0.805, *P* = 0.005). The results of internal validation and external validation proved the robustness of our proposed approach.

**Figure 4:**
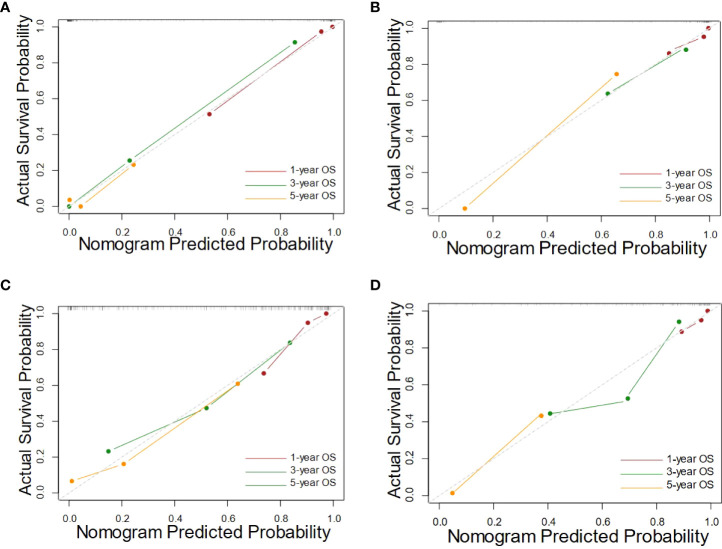
The calibration curves of the integrated nomograms in training cohort **(A)** and the validation cohort **(B)**, along with the clinical nomograms in training cohort **(C)** and the validation cohort **(D)**.

The calibration curves of nomograms were plotted in **Figure 4**. The calibration curves showed good consistent between the training cohort and the validation cohort, and the calibration curve of the integrated nomogram also showed better performance.

## Discussion

In this study, we proposed an automated approach for predicting glioma grade and survival of LGG patients using CNN and radiomics. The segmentation network was used to generate ROI masks for radiomics features extraction in an automated pipeline and assisting the grading network to focus on the tumor region without requiring the radiologist to manually delineate the tumor region. ICCs were used to evaluate the consistency between results of segmentation network and ground truth annotated by radiologists in feature extraction. In survival analysis, a deep-radiomics signature was developed as a novel prognostic factor to predict OS of LGG patients. Then, we developed an integrated nomogram, which was constructed by incorporating the deep-radiomics signature and clinical prognostic factors and independently validated in the internal and external cohort.

CNN and radiomics analysis are representative quantitative methods for image analysis, which can extract high-dimensional and abstract numeric information beyond what is perceivable *via* the visual assessment of a given image (15). Multiple studies have focused on the grading and survival analysis of glioma on preoperative multimodal images using radiomics or deep learning methods (29, 52–57). Although related studies using CNN or radiomics methods have shown admirable performance, both two methods have encountered many difficulties when it comes to clinical practice. First, the reproducibility of mask extraction is critical when analyzed by CNN or radiomics method. In the related studies, especially in radiomics-based survival analysis, masks were delineated manually by radiologists, which required expensive time and labor costs. Semi-supervised segmentation has also been applied in tumor grading in recent years, but a fully automatic segmentation is still an urgent need to achieve ideal reproducibility. Second, CNN is an end-to-end training network that obtains key information through convolution operation and often has excellent performance, but its interpretability in feature extraction and selection is not relatively good. Third, medical images are usually provided in a three-dimensional format, 3D CNN network can fully obtain the 3D shape and position information of the tumor, but the construction of the 3D CNN network requires a large number of images to train the network, so few studies use the 3D network to analyze the glioma. A few studies have attempted to combine CNN with radiomics for gliomas grading. One study with a total of 252 patients developed an approach based on the combination of radiomics features and 2D CNN features from multiplanar reconstructed (MPR) images, which achieved the highest AUC of 0.874 (58). Although this approach has shown good performance, the masks used for feature extraction were manually delineated, and the input of the CNN were the slices with the largest tumor area and the two adjacent images, which failed to provide the information of tumor comprehensively. Another study designed a fully automatic process including segmentation and grading, they selected 5 MRI slices of each patient as the input of 2D CNN and added the shape and site radiomics features to the fully connected layer to participate in the training of CNN (59). However, the hybrid model failed to explain improved performance compared to the CNN model only. Radiomics features may be dropped during the training, because authors cannot monitor the specific process of CNN training. In the current study, given that the limitation of the unbalanced patient categories, we attempted to randomly assign the patients and merged the radiomics features with the CNN features based on 3D network. Then, we applied the machine learning algorithm to reduce feature dimensions, which can not only visualize the process of feature selection, but also make full use of 3D CNN to extract spatial information of tumor. Through ablation experiments, the combination of radiomics features and features extracted from patches-based 3D CNN showed better performance than other methods.

Radiomics has also been applied in the survival analysis of glioma patients (28, 29, 54). However, to the best of our knowledge, there are few glioma prognosis models based on CNN methods. This may be due to the fact that CNN is an end-to-end model, where the image features are iteratively calculated in the network and cannot be intuitively visualized by researchers. Hence, it is difficult to train a CNN model that can predict patient survival on a limited dataset. In our study, the deep-radiomics signature developed by radiomics and CNN was used for the first time in the survival analysis of glioma, which enabled high-throughput and abstract digital information to be clearly recorded in feature extraction and selection, successfully made full use of deep learning and radiomics features to compensate for the imperfect interpretability of CNN. It is worth noting that the classification-guided prediction strategy was used on CNN to supervise the efficiency of learning, and the useful information was mined as fully as possible by sharing weights between the classification task and the regression task. In related studies, one study has developed a radiomics signature based on T2WI and FLAIR sequences for predicting overall survival of LGG patients, with a C-index of 0.763 in the validation cohort (54). Another study with 233 patients has developed a radiomic risk score based on T2WI sequence using linear calculation to predict prognosis in LGG patients (60). Nonetheless, the pipelines of those studies are still limited by some features and are not automated. Compared with those two studies, we demonstrated the features from T1-Gd sequence were better than those of T1WI and T2WI sequences in predicting the survival of LGG patients. In the current study, the pipeline of survival analysis was automated without masks annotated by radiologist. We analyzed the difference between the features extracted from delineation by radiologists and the features extracted from the result of segmentation network to validate the reproducibility of the fully automatic segmentation. Moreover, it is worth noting that our signature was validated in a new independent center, which ensured the robustness of the model.

There are four main limitations of this study that are worth discussing. First, although the patients enrolled in this study are from two independent centers, it is necessary to enroll more patients and more multiple centers to validate our proposed approach. Second, our study is based on conventional structural MRI images and does not use multimodal images for feature extraction due to avoiding feature redundancy and data limitation. Our approach may achieve better performance if we add functional MRI images in this study. Third, molecular subtypes are considered as an important factor, which can reflect the prognosis of tumors to some extent. However, most hospitals in China have not performed clinical molecular diagnosis for LGG patients. Therefore, the molecular diagnosis result was not available in our study. In addition, relatively large batch size may further improve the performance of the segmentation network, but the limited computing resources make it difficult to use large batch sizes at present. In future work, we will explore the impact of batch size on the segmentation performance and improve 3D-UNet for better segmentation performance, such as introducing attention mechanism.

## Conclusion

In this study, we developed a fully automated approach combining CNN with radiomics in glioma grading and prognostic analysis of LGG patients. Our proposed approach can be easily integrated into the clinical setting that can be widely used as a practical tool to facilitate patient diagnosis, individualized treatment planning, and prognostic assessment without additional healthcare expenses.

## Data availability statement

The original contributions presented in the study are included in the article/**Supplementary Material**. Further inquiries can be directed to the corresponding authors.

## Ethics statement

The studies involving human participants were reviewed and approved by Institutional Review Board of Jinling Hospital, Medical School of Nanjing University, China. Informed consent was waived because of the use of retrospective image data.

## Author contribution

All authors contributed to the article and approved the submitted version. CX designed the study, conducted most of experiments, analyzed the experimental results, and drafted the manuscript. YP and WZ reviewed and revised the manuscript. ZC and WT reviewed the manuscript and gave professional advice. JL collected, annotated and interpreted the experimental data in this study. ZZ designed the study, annotated the experimental data, and provided guidance for clinical data analysis. XC designed the study, gave insight in experimental design, reviewed and revised the manuscript.

## Funding

This study was supported in part by the National Key R&D Program of China (2018YFA0701700) and part by the National Nature Science Foundation of China (U20A20170, 61622114).

## Conflict of interest

The authors declare that the research was conducted in the absence of any commercial or financial relationships that could be construed as a potential conflict of interest.

## Publisher’s note

All claims expressed in this article are solely those of the authors and do not necessarily represent those of their affiliated organizations, or those of the publisher, the editors and the reviewers. Any product that may be evaluated in this article, or claim that may be made by its manufacturer, is not guaranteed or endorsed by the publisher.
