# Supplementary material for: An automated approach for predicting glioma grade and survival of LGG patients using CNN and radiomics
Source: Front Oncol. 2022 Aug 12;12:969907. doi: 10.3389/fonc.2022.969907 (PMC9413530; doi:10.3389/fonc.2022.969907)
Supplement: Supplementary file 1 [file DataSheet_1.pdf]

Supplementary Material

1 Supplementary Data

S1. MRI acquisition parameters

MRI scans were acquired using 3.0 T scanner (Siemens, TrioTim), including axial T1-weighted images (repetition time, 2300ms; echo time, 2.98ms; slice thickness, 1mm), and T1-Gd images were acquired using 0.1 mmol/kg of Gd-DTPA injections (Beijing Beilu Pharmaceutical Co.; repetition time, 2350ms; echo time, 2.98ms; slice thickness, 1mm), and T2-weighted images (repetition time, 4500ms; echo time, 84ms; slice thickness, 1mm), with a field of view 256 mm × 256mm, and a matrix size of 256 × 256 pixels.

S2. Radiomics features

In this study, radiomics features were obtained by extracting high-throughput features from region of interest (ROI) in MRI images, including shape features, first-order features, texture features and wavelet features. The extracted features and their effects are as follows:

(1) First-order features (n=18) describe the distribution of gray intensities within the image region defined by the ROI, which can reflect the heterogeneity within the tumor.
(2) Shape and size features (n=14) quantitatively describe the geometric properties of the ROI, such as tumor surface area, volume, surface area to volume ratio, sphericity, compactness, and three-dimensional diameter, etc. These features are independent from the gray level intensity distribution in the ROI and only calculated on the non-derived image and mask, which can describe the size and shape of the tumor in three dimensions.
(3) Texture features (n=68) reflect information about the spatial arrangement of voxel intensities. They are derived from four statistical feature matrices: 22 from the Gray-level co-occurrence matrix (GLCM), 16 from the gray level size zone matrix (GLSZM), 16 from the Gray-level run-length texture matrix (GLRLM) and 14 from the gray level dependence matrix (GLDM). These features can quantify information such as texture patterns or tissue distribution within the tumor that are difficult to simply perceive visually.
(4) Wavelet features (n=688) contain high-dimensional information of the images. Eight wavelet-transformed images are generated after 3D wavelet decomposition of the original MRI image through directional low-pass and high-pass filtering. Then, the first-order and texture features are extracted from the eight wavelet-transformed images of each original MRI image respectively, resulting in a total of 688 features, i.e., (18+68) features/image × 8 images. Wavelet transform can obtain multi-frequency domain and multi-scale image information. For clinical problems that are difficult to describe with simple visual features of tumor images, wavelet features can play different roles and capture clinical information that is not easily perceived by visual perception.

2 Supplementary Figures and Tables

2.1 Supplementary Figures

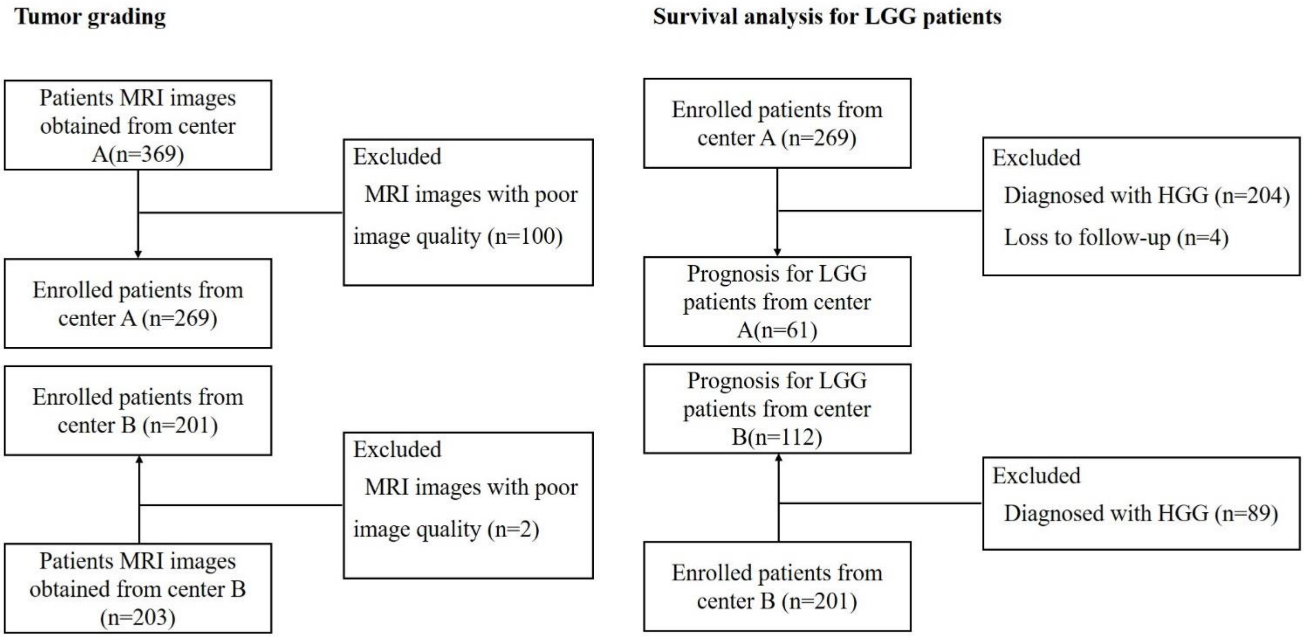

Figure S1 | Enrollment pathway

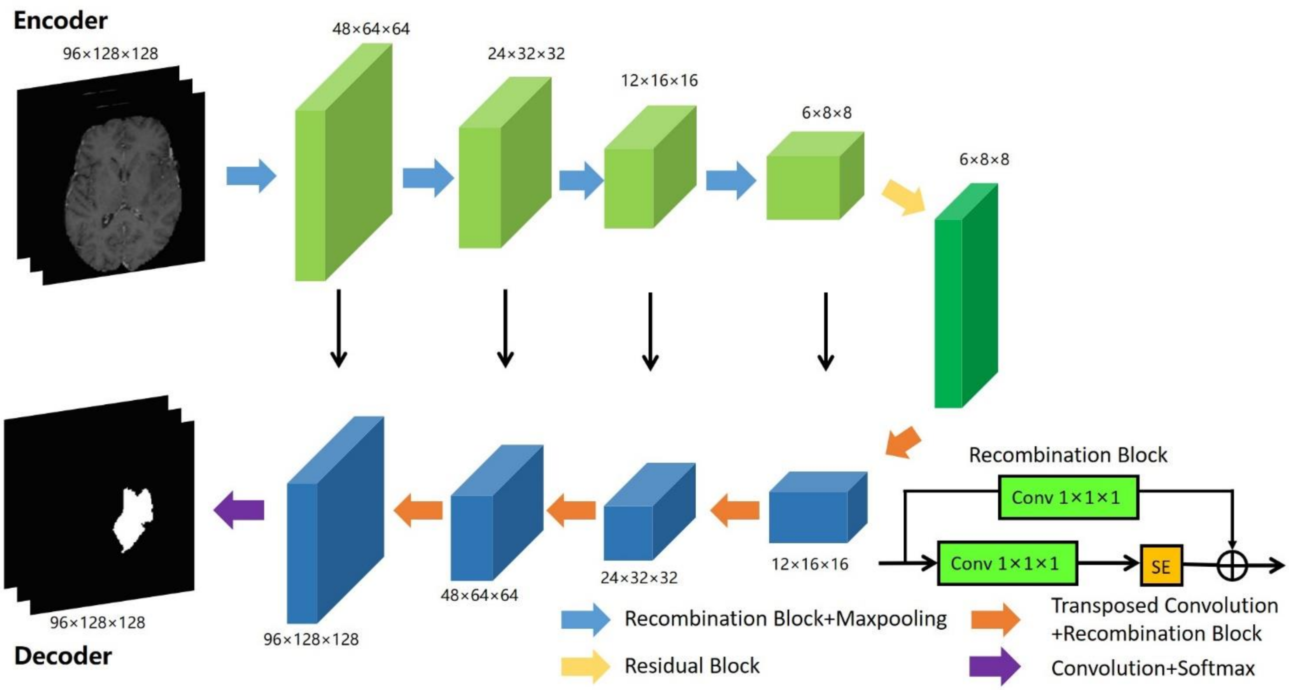

Figure S2 | Network architecture of tumor segmentation

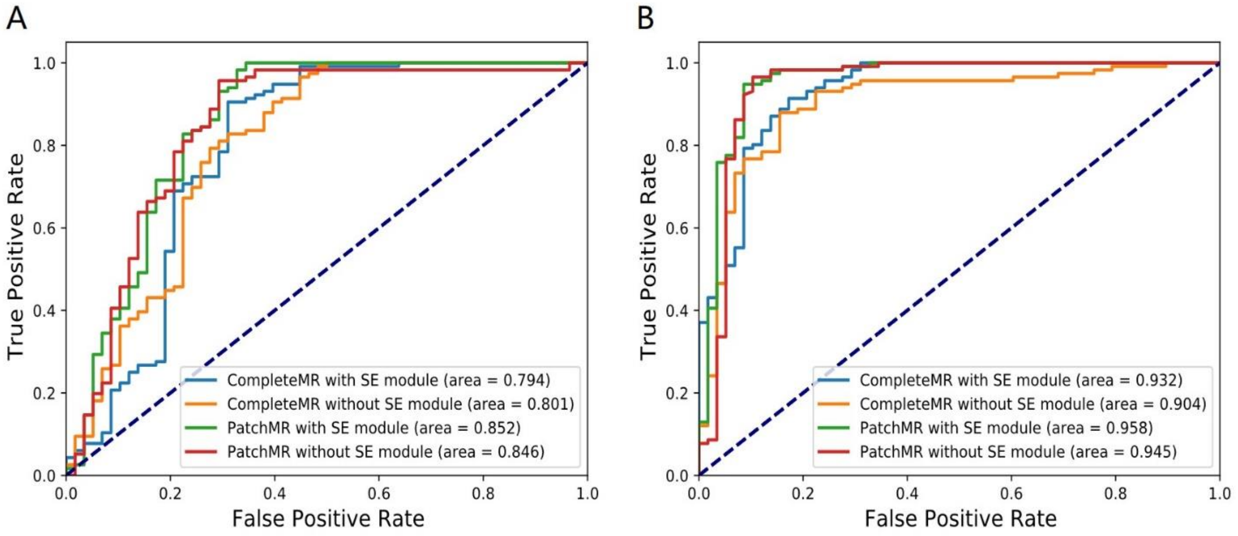

Figure S3 | The Receiver Operating Characteristic(ROC) Curve of the grading based on the CNN only(A) and the grading based on the integrated approach(B).

2.2 Supplementary Tables

Table S1 | List of radiomic features extracted from Pyradiomics

Feature category	Feature list
First order (n=18)	Energy, Total Energy, Entropy, Minimum, 10th percentile, 90th percentile, Maximum, Mean, Median, Interquartile Range, Range, Mean Absolute Deviation, Robust Mean Absolute Deviation, Root Mean Squared, Skewness, Kurtosis, Variance, Uniformity
Shape and size (n=14)	Mesh Volume, Voxel Volume, Surface Area, Surface Area to Volume ratio, Sphericity, Maximum 3D diameter, Maximum 2D diameter(Slice), Maximum 2D diameter(Column), Maximum 2D diameter(Row), Major Axis Length, Minor Axis Length, Least Axis Length, Elongation, Flatness
GLCM (n=22)	Autocorrelation, Joint Average, Cluster Prominence, Cluster Shade, Cluster Tendency, Contrast, Correlation, Difference Average, Difference Entropy, Difference Variance, Joint Energy, Joint Entropy, Informal Measure of Correlation 1, Informal Measure of Correlation 2, Inverse Difference Moment, Inverse Difference Moment Normalized, Inverse Difference, Inverse Difference Normalized, Inverse Variance, Maximum Probability, Sum Entropy, Sum of Squares
GLSZM (n=16)	Small Area Emphasis, Large Area Emphasis, Gray Level Non-Uniformity, Gray Level Non-Uniformity Normalized, Size-Zone Non-Uniformity, Size-Zone Non-Uniformity Normalized, Zone Percentage, Gray Level Variance, Zone Variance, Zone Entropy, Low Gray Level Zone Emphasis, High Gray Level Zone Emphasis, Small Area Low Gray-Level Emphasis, Small Area High Gray-Level Emphasis, Large Area Low Gray-Level Emphasis, Large Area High Gray-Level Emphasis
GLRLM (n=16)	Short Run Emphasis, Long Run Emphasis, Gray Level Non-Uniformity, Gray Level Non-Uniformity Normalized, Run Length Non-Uniformity, Run Length Non-Uniformity Normalized, Run Percentage, Gray Level Variance, Run Variance, Run Entropy, Low Gray Level Run Emphasis, High Gray Level Run Emphasis, Short Run Low Gray-Level Emphasis, Short Run High Gray-Level Emphasis, Long Run Low Gray-Level Emphasis, Long Run High Gray-Level Emphasis
GLDM (n=14)	Small Dependence Emphasis, Large Dependence Emphasis, Gray Level Non-Uniformity, Dependence Non-Uniformity, Dependence Non-Uniformity Normalized, Gray Level Variance, Dependence Variance, Dependence Entropy, Low Gray-Level Emphasis, High Gray-Level Emphasis, Small Dependence Low Gray-Level Emphasis, Small Dependence High Emphasis, Large Dependence Low Gray-Level Emphasis, Large Dependence High Gray-Level Emphasis
Wavelet (n=688)	Features extracted from 8 levels of LLL,LLH,LHH,HHH,HHL,HLH,LHL,HLL

Note: GLCM: Gray Level Co-occurrence Matrix; GLSZM: Gray Level Size Zone Matrix; GLRLM: Gray Level Run Length Matrix; NGTDM: Neighboring Gray Tone Difference Matrix; GLDM: Gray Level Dependence Matrix

Table S2 | The clinical characteristics of the training and validation cohorts

Characteristic	Level	Training cohort (n=112)	Validation cohort (n=61)	*P*
Gender	Male	52 (46.4)	26 (42.6)	0.631
	Female	60 (53.6)	35 (57.4)	
WHO grade	Grade II	49 (43.8)	19 (31.1)	0.142
	Grade III	63 (56.2)	42 (68.9)	
Laterality	Left	51 (45.5)	28 (45.9)	0.963
	Right	61 (54.5)	33 (54.1)	
Tumor location	Frontal lobe	59 (52.7)	32 (52.5)	0.461
	Temporal lobe	33 (29.5)	14 (23.0)	
	Parietal lobe	10 (8.9)	4 (6.6)	
	Occipital lobe	5 (4.5)	6 (9.8)	
	Insular lobe	5 (4.5)	5 (8.2)	
Histologic	Astrocytoma	62 (55.4)	30 (49.2)	0.332
	Oligodendroglioma	40 (35.7)	21 (34.4)	
	Oligoastrocytoma	10 (8.9)	10 (16.4)	
Contrast	Not enhanced	52 (46.4)	36 (59.0)	0.152
	Enhanced	60 (53.6)	25 (41.0)	
Age	Mean ± SD	47.4±12.2	44.3±12.8	0.175
OS (months)	Mean ± SD	41.4±30.9	33.4±30.1	0.601

Note: Numbers in parenthesis are percentage; WHO: World Health Organization; OS: overall survival; SD: standard deviance

Table S3 | The AUC, accuracy, sensitivity, and precision of the radiomics grading models

Sequence	AUC	Accuracy	Sensitivity	Precision
T1 weighted images	0.873 (0.867,0.879)	0.876	0.923	0.832
T1-Gd weighted images	0.894 (0.892,0.896)	0.886	0.931	0.875
T2 weighted images	0.829 (0.825,0.833)	0.801	0.843	0.781

Table S4 | The features and corresponding weights in developing the deep-radiomics signature

Feature	Weight
Deep CNN feature-74	0.1416
Deep CNN feature -254	0.2178
Deep CNN feature -273	0.3161
Deep CNN feature -289	0.2117
Deep CNN feature -274	0.2388
Deep CNN feature -36	0.5766
Wavelet.LLL_glszm_SmallAreaHighGrayLevelEmphasis	0.4943
Wavelet.LHH_glrlm_LowGrayLevelRunEmphasis	0.4467
Original_gldm_GrayLevelNonUniformity	0.5765
Wavelet.LHL_glszm_GrayLevelNonUniformityNormalized.	0.5996

Note: GLSZM: Gray Level Size Zone Matrix; GLRLM: Gray Level Run Length Matrix
